# Analysis of the role of COP9 Signalosome (CSN) subunits in K562; the first link between CSN and autophagy

**DOI:** 10.1186/1471-2121-10-31

**Published:** 2009-04-28

**Authors:** Claire Pearce, Rachel E Hayden, Christopher M Bunce, Farhat L Khanim

**Affiliations:** 1 College of Life and Environmental Sciences, School of Biosciences, University of Birmingham, Edgbaston, Birmingham, B15 2TT, UK

## Abstract

**Background:**

The COP9/signalosome (CSN) is a highly conserved eight subunit complex that, by deneddylating cullins in cullin-based E3 ubiquitin ligases, regulates protein degradation. Although studied in model human cell lines such as HeLa, very little is known about the role of the CSN in haemopoietic cells.

**Results:**

Greater than 95% knockdown of the non-catalytic subunit CSN2 and the deneddylating subunit CSN5 of the CSN was achieved in the human myeloid progenitor cell line K562. CSN2 knockdown led to a reduction of both CSN5 protein and mRNA whilst CSN5 knockdown had little effect on CSN2. Both knockdowns inhibited CSN deneddylase function as demonstrated by accumulation of neddylated Cul1. Furthermore, both knockdowns resulted in the sequential loss of Skp2, Cdc4 and β-TrCP F-box proteins. These proteins were rescued by the proteasome inhibitor MG132, indicating the autocatalytic degradation of F-box proteins upon loss of CSN2 or CSN5. Interestingly, altered F-box protein gene expression was also observed in CSN2 and CSN5 knockdowns, suggesting a potential role of the CSN in regulating F-box protein transcription.

Loss of either CSN subunit dramatically reduced cell growth but resulted in distinct patterns of cell death. CSN5 knockdown caused mitotic defects, G2/M arrest and apoptotic cell death. CSN2 knockdown resulted in non-apoptotic cell death associated with accumulation of both the autophagy marker LC3-II and autophagic vacuoles. Treatment of vector control K562 cells with the autophagy inhibitors 3-methyladenine and bafilomycin A1 recapitulated the growth kinetics, vacuolar morphology and LC3-II accumulation of CSN2 knockdown cells indicating that the cellular phenotype of CSN2 cells arises from autophagy inhibition. Finally, loss of CSN2 was associated with the formation of a CSN5 containing subcomplex.

**Conclusion:**

We conclude that CSN2 is required for CSN integrity and the stability of individual CSN subunits, and postulate that CSN2 loss results in a phenotype distinct from that of cells lacking CSN5 possibly as a consequence of altered CSN5 activity within a resultant CSN subcomplex. Our data present the first evidence for the sequential loss of F-box proteins upon CSN manipulation and are the first to identify a potential link between CSN function and autophagy.

## Background

The regulated expression and degradation of proteins are critical to all aspects of cell development and proliferation. The two main routes for eukaryotic intracellular protein clearance are the ubiquitin-proteasome system (UPS) and the autophagy-lysosome pathway. A key component involved in regulating degradation of proteins by the UPS is the COP9 signalosome (CSN). The CSN is an eight-subunit (CSN1-8) protein complex, highly conserved amongst eukaryotes [1-5] originally identified in Arabidopsis as a negative regulator of photomorphogenesis [6]. Through its function in the regulation of the UPS, the CSN has been implicated in the regulation of biological processes as diverse as DNA replication and repair, cell-cycle progression and cell development [7-9].

Degradation of cellular proteins by the 26S proteasome [10-13] is preceded by ubiquitination of target proteins [14], a process mediated by three enzyme complexes; a ubiquitin activating enzyme (E1), a ubiquitin conjugating enzyme (E2) and a ubiquitin ligase (E3) [15]. The E3 ligase interacts with the protein substrate and thus confers the specificity of the UPS [16]. The largest known class of E3 ubiquitin ligases comprises the Cullin-RING ligases (CRLs) of which the best studied is the SCF (Skp1, Cul1, F-box protein) complex [16]. The cullin subunit (Cul1) of the SCF forms a scaffold to recruit and bring into close proximity the E2 and its substrate, thereby facilitating ubiquitin transfer from the E2 to target proteins (SCF structure reviewed in [16]). The RING protein (Hrt1/Roc1/Rbx1) is the fourth subunit of the SCF and is responsible for E2 recruitment, whilst the variable F-box protein subunit, recruited to the SCF complex via the adaptor protein Skp1, binds substrates selectively [17-19]. In yeast, over 19 F-box proteins are known, over 400 in A. thaliana, and ~70 in humans [16]. Since each cullin (Cul1-5) forms complexes with a variable substrate recognition subunit (SRS) (F-box proteins for Cul1 as above, VHL box proteins for Cul2, BTB proteins for Cul3, WD40 proteins for Cul4 and SOCS box proteins for Cul5, reviewed in [20]) specificity in CRL target protein recruitment is achieved by the large number of variable SRS containing CRLs. It is thought that, altogether, the human genome may have the capacity to code for as many as 350 different CRLs.

Given the potential number and diversity of target proteins requiring CRL mediated ubiquitination for degradation, dynamic regulation of the CRL complex repertoire in a cell at any given time is essential. All cullins studied (Cul1-5) have been shown to be modified by neddylation [21], which facilitates their ubiquitin ligase activity [22] possibly via increased E2 affinity [23,24]. The deneddylation of cullins is mediated by the CSN complex [25]. Although initial studies indicated a negative role for deneddylation, further studies have implicated deneddylation in the positive regulation of CRL activity [3,4,26]. It has since been proposed by several groups that optimal CRL activity requires the cyclic neddylation and deneddylation of the cullin subunit [4,7,27]. Although the exact mechanisms are not fully understood, it is thought that F-box proteins themselves are targeted for degradation in part by autoubiquitination within the SCF complex [28]. The deneddylation of cullins by the CSN is believed to regulate the autoubiquitination of SRSs [28,29], thereby modulating CRL composition and activity. Furthermore, the CSN has been shown to be associated with a deubiquitinase activity which may further stabilize autoubiquitinated SRSs [27,29]. The CSN complex is therefore an integral regulator of CRL activity and subsequent protein degradation.

In this present study we have investigated the effects of knocking down CSN2 and CSN5 in the model K562 cell line, a model of human erythrocyte and megakaryocyte progenitors [30,31]. Whilst knockdown of either CSN2 or CSN5 resulted in common changes including the sequential loss of F-box proteins Skp2, Cdc4 and β-TrCP other important differences occurred. For example CSN5 knockdown resulted in apoptotic cell death associated with aberrant mitosis, whereas CSN2 knockdown cells underwent non-apoptotic cell death that was associated with both inhibition of autophagy and the formation of a novel CSN5 containing CSN subcomplex.

## Results

### CSN2 and CSN5 knockdown and resulting aberrant SCF activity

**Figure 1:**
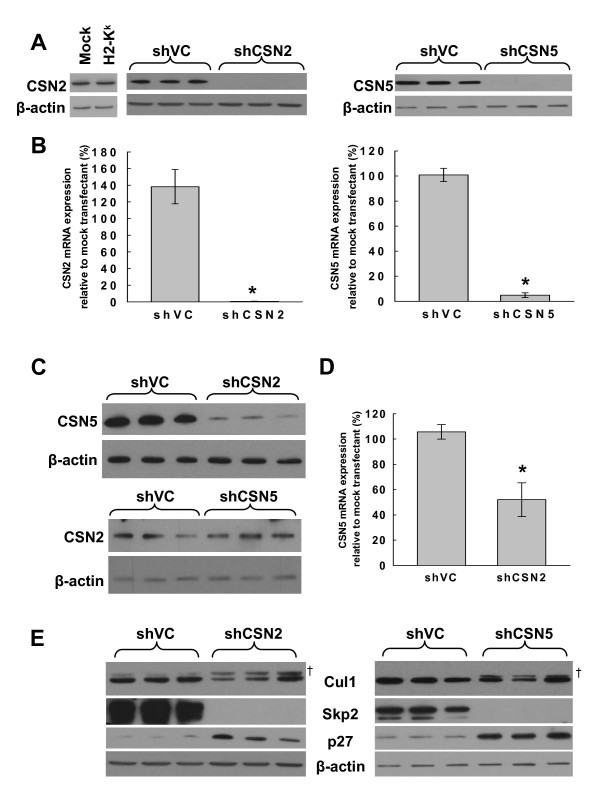
**CSN2 and CSN5 knockdowns result in Cul-1 hyperneddylation, loss of Skp-2 and accumulation of p27**. K562 cells were transiently transfected in the absence of plasmid DNA (mock), with pMACS K^k^.II plasmid alone (HKK) or transiently co-transfected with HKK plasmid together with either vector control (shVC), CSN2 knockdown (shCSN2) or CSN5 knockdown (shCSN5) plasmid. HKK positive cells were sorted 24 hours post-transfection, re-cultured and harvested for analysis. (A) CSN2 (left) and CSN5 (right) protein knockdown was determined by western blot in three independent transfectants. (B) mRNA levels in vector control and knockdown cells were determined by QRT-PCR (CSN2, left, P = 0.0012; CSN5, right, P = 0.000031). Data represents 3 independent sets of triplicate transfections. (C) CSN5 and CSN2 protein levels were determined by western blot in n = 3 CSN2 (top) and CSN5 (bottom) knockdowns, respectively. (D) CSN5 mRNA levels in n = 3 vector control and CSN2 knockdown cells were determined by QRT-PCR (P = 0.011). (E) The level of Cul1 neddylation (top panels, ^† ^indicates neddylated Cul-1), Skp2 protein (second panels) and p27 protein (third panels) was determined in n = 3 shCSN2 (left) and shCSN5 (right) samples by western blot. Even gel loading was determined by β-actin signal. Graphical data indicates the mean ± s.e.m. * indicates significance with a p value of less than 0.05.

Plasmids encoding short hairpin RNA (shRNA) to either CSN2 or CSN5 were electroporated into K562 cells together with H-2K^k ^plasmid and transfected cells sorted by anti- H-2K^k ^magnetic beads. Three days post transfection, neither CSN2 nor CSN5 protein could be detected by western blot analysis in their respective knockdown protein extracts (Fig. 1A). Furthermore, CSN2 knockdown of over 99% and CSN5 knockdown of over 95% was achieved at the mRNA level relative to mock transfectants as measured by quantitative real-time PCR (QRT-PCR) (Fig. 1B). This level of knockdown was maintained throughout the time course of the experiments (data not shown). Transfection with either the H-2K^k ^plasmid alone or co-transfection with H-2K^k ^plasmid and empty vector (shVC) had no effect on the level of CSN2 or CSN5 mRNA or protein relative to mock transfected cells (Fig. 1A &1B and data not shown). Co-transfections with H-2K^k ^and a vector encoding a control scrambled shRNA sequence had no effect on the level of CSN2 or CSN5 mRNA or protein relative to cells co-transfected with empty vector (Additional file 1), demonstrating that the results were specific and not due to off-target effects.

The instability of particular CSN subunits in the absence of another subunit has been reported previously. For example, CSN8 knockdown has been shown to result in the loss of CSN3, CSN5 and CSN7 protein [32]. We therefore determined CSN5 and CSN2 protein levels in CSN2 and CSN5 knockdown cells, respectively, by western blotting. A significant reduction in CSN5 protein was observed in cells lacking CSN2, whilst depletion of CSN5 had no effect on CSN2 protein or mRNA levels relative to vector control cells (Fig. 1C and data not shown). Interestingly, CSN2 knockdown not only resulted in loss of CSN5 protein, but also in the significant reduction of CSN5 mRNA as determined by QRT-PCR (Fig. 1D, P = 0.011).

Consistent with previous studies of CSN deregulation [28,33], both CSN2 and CSN5 knockdowns were associated with accumulation of neddylated Cul1 (Fig. 1E) indicating functional dysregulation of the CSN following knockdown of either subunit. Studies demonstrating Cul1 hyperneddylation in CSN deficient cells have also demonstrated degradation of the F-box protein Skp2 [28,33]. In agreement with these observations, complete loss of Skp2 protein was observed in both the CSN2 and CSN5 knockdown cells (Fig. 1E). Skp2 binds to and mediates the ubiquitination of multiple proteins, including the cyclin dependent kinase inhibitor p27^kip1 ^[34]. Consistent with loss of Skp2, knockdown of CSN2 and CSN5 resulted in the accumulation of p27 (Fig. 1E).

### Loss of CSN2 and CSN5 results in the sequential loss of F-box proteins

**Figure 2:**
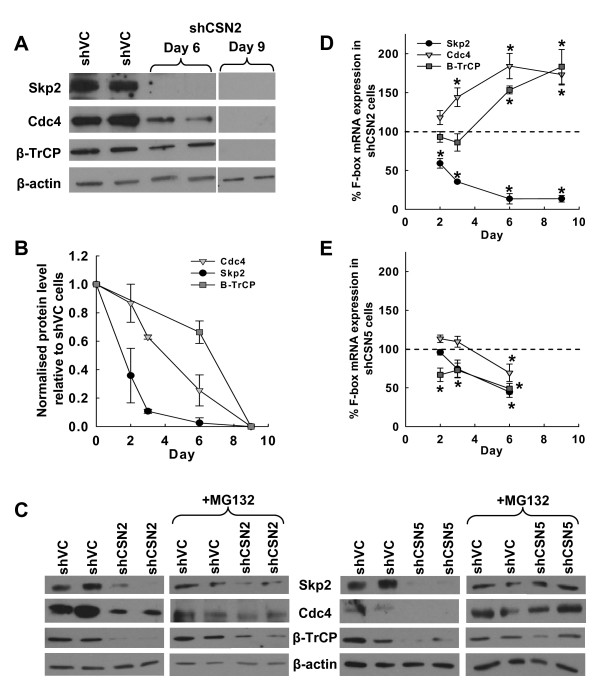
**Loss of CSN2 and CSN5 results in sequential loss of F-box proteins**. K562 cells were transiently co-transfected with HKK plasmid together with either shVC, shCSN2 or shCSN5 plasmid. HKK positive cells were sorted 24 hours post-transfection and cells re-cultured. (A) shVC and shCSN2 cells were harvested day 6 and 9 post transfection and the level of Skp2, Cdc4, β-TrCP and β-actin protein determined by western blot. (B) The level of Skp2, Cdc4 and β-TrCP proteins (normalised for loading using β-actin) in shCSN2 at each time point was normalised to expression in shVC cells and the data plotted as the mean ± s.e.m. (C) shVC, shCSN2 and shCSN5 cells were treated with DMSO (control) or the proteasome inhibitor MG132 (10 μM) for the final 18 hours of culturing and the level of Skp-2, Cdc4, β-TrCP and β-actin protein determined by western blot. (D, E) The level of Skp2, Cdc4 and β-TrCP mRNA in shCSN2 (D) and shCSN5 (E) cells was determined at each time point post transfection relative to expression in vector control scramble cells by QRT-PCR. Data shown is the mean ± s.e.m of n = 3 transfections. * indicates a significant difference to vector controls with a p value of less than 0.05.

Skp2 is one of many F-box proteins that can bind to the Cul1/Roc1/Skp1 complex, thereby altering the substrate specificity of the SCF (F-box proteins reviewed in [19]). Analysis of three such F-box proteins, Skp2, Cdc4 and β-TrCP revealed a sequential loss of protein in CSN2 and CSN5 knockdown cells (Fig. 2A &2B and Additional file 1, part D). In CSN2 knockdown cells, Skp2 protein was reduced by ~60% by day 2 and over 90% by day 6, whereas Cdc4 was lost at a slower rate with 30–40% lost by day 3 increasing to ~70% by day 6 (Fig. 2B). Loss of β-TrCP was still more retarded with ~70% protein remaining at day 6 (Fig. 2B). By day 9, all three F-box proteins were undetectable in CSN2 knockdown cells (Fig. 2A). Similar sequential loss of F-box proteins was observed in CSN5 knockdown cells. At day 4 post transfection Skp2 protein could not be detected, Cdc4 protein was greatly reduced but remained detectable, and β-TrCP was largely unaffected (Additional file 1, part D).

In order to determine the mechanism of F-box protein loss in both CSN2 and CSN5 knockdown cells, we investigated the rescue of F-box proteins by the proteasome inhibitor MG132 (Fig. 2C) and measured the levels of F-box mRNA over time (Fig. 2D &2E). Treatment of both CSN2 and CSN5 knockdown cells with MG132 resulted in Skp2, Cdc4 and β-TrCP protein rescue (Fig. 2C), indicating involvement of the proteasome in the loss of these F-box proteins. However the strength of the observed rescue varied. Importantly, CSN subunit depletion also affected levels of F-box mRNA. CSN2 knockdown resulted in a significant reduction of Skp2 mRNA by day 2 post transfection. Thus it is likely that transcriptional changes also contribute to loss of Skp2 protein in these cells. In contrast Cdc4 and β-TrCP mRNAs were significantly increased following CSN2 knockdown despite clear loss of protein (Fig. 2D). CSN5 depletion resulted in a modest reduction of all three F-box protein mRNAs but not enough to account for the observed loss of protein (Fig. 2E). Collectively these data indicate that, with the possible exception of Skp2 in CSN2 knockdown cells, proteasomal degradation rather than transcriptional repression was the main driver of F-box protein loss in CSN2 and CSN5 knockdown K562 cells.

### Both CSN2 and CSN5 knockdowns result in reduced cell growth and cell death

**Figure 3:**
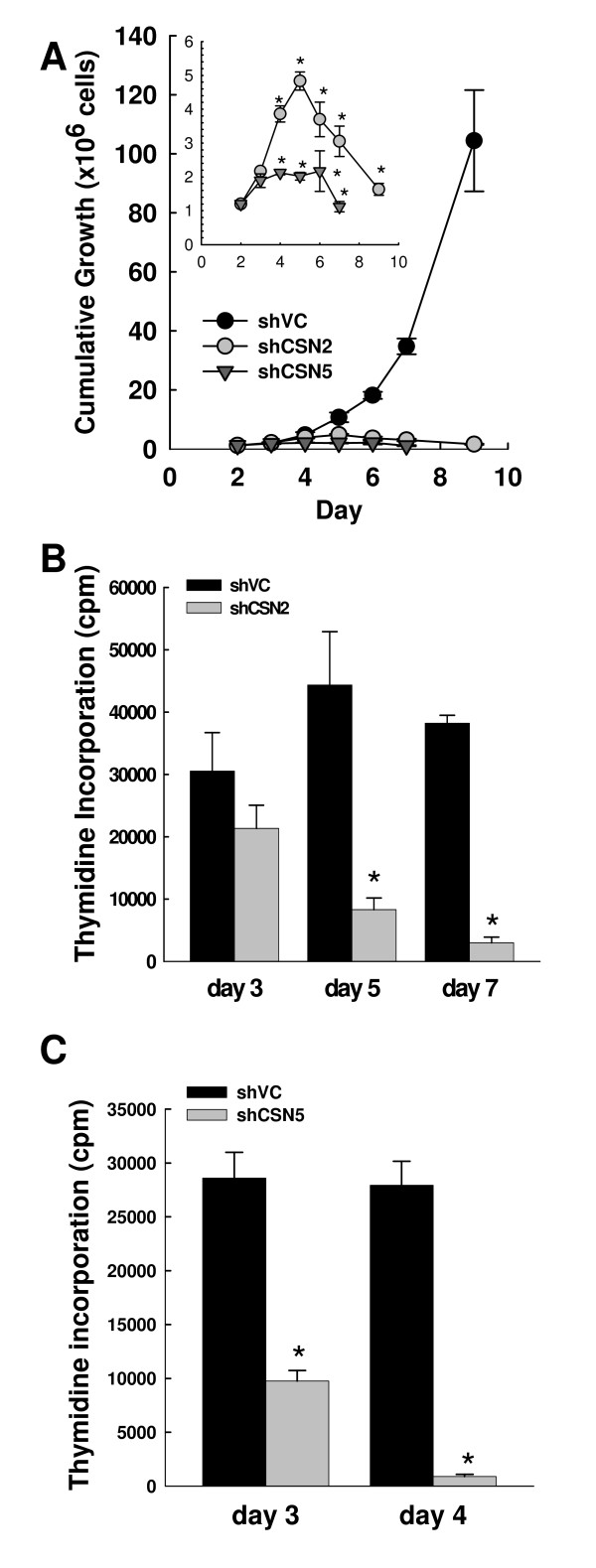
**Both CSN2 and CSN5 knockdowns result in reduced cell growth**. K562 cells were transiently co-transfected with HKK plasmid together with either shVC, shCSN2 or shCSN5 plasmid. HKK positive cells were sorted 24 hours post-transfection and cells re-cultured. (A) Cell counts were taken daily and the cumulative growth calculated. The cumulative growth of shCSN2 and shCSN5 cells is shown relative to shVC. The insert graph has a different Y scale to highlight the differences between shCSN2 and shCSN5 cumulative growth profiles. Data shown are the mean ± s.e.m. of n = 3. * indicates a significant difference to shVC cell growth with a p value of less than 0.05. (B) Thymidine incorporation in shCSN2 cells relative to shVC cells was measured day 3, 5 and 7 post transfection. Data shown are the mean ± s.e.m. of n = 3. (C) Thymidine incorporation in shCSN5 cells relative to shVC cells was measured day 3 and 4 post transfection. Data shown are the mean ± s.e.m. of n = 3. * indicates significance with a p value of less than 0.05.

Knockdown of either CSN2 or CSN5 dramatically diminished cell proliferation, followed by loss of cell viability (Fig. 3A). Analysis of cumulative growth demonstrated that proliferation of cells lacking CSN2 was significantly reduced when compared to that of shVC cells by day 4 post transfection (Fig. 3A, P = 0.034), whilst loss of proliferation following CSN5 knockdown was even more marked (Fig. 3A insert, P = 0.0023). This difference in diminished proliferation was corroborated by measurement of thymidine incorporation. At day 3 post-transfection, there was no significant difference in the incorporation of tritiated thymidine into cellular DNA between shVC and CSN2 knockdown (Fig. 3B) whereas the CSN5 knockdown cells already had markedly reduced thymidine incorporation (Fig. 3C, P = 0.0012). However, by day 5 the CSN2 knockdown cells also had a significant decrease in thymidine incorporation (Fig. 3B, P = 0.0072) which decreased even further by day 7 (P = 1.1 × 10^-5^). Thus, although the changes in Cul1 neddylation and F-box protein levels are very similar between the CSN2 and CSN5 knockdowns, differences were apparent in the growth kinetics of the cells.

### Knockdown of CSN5 but not CSN2 is associated with cell cycle arrest and defects in mitotic spindle formation

**Figure 4:**
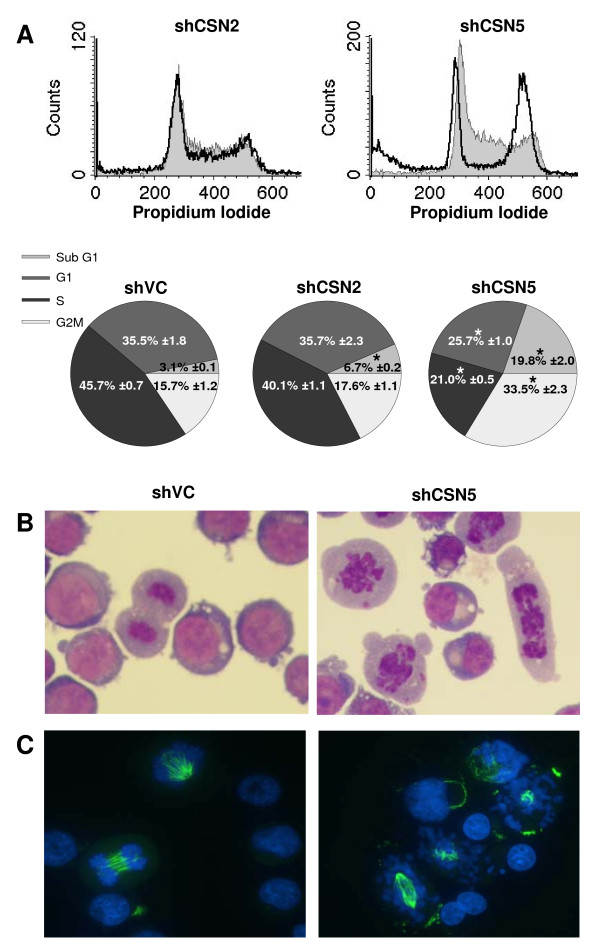
**Cell cycle arrest in CSN5 knockdowns is associated with mitotic defects**. K562 cells were transiently co-transfected with HKK plasmid together with shVC, shCSN2 or shCSN5 plasmid. HKK positive cells were sorted 24 hours post-transfection and cells re-cultured. (A) Representative images of shCSN2 (left, dark grey line) and shCSN5 (right, dark grey line) cell cycle profiles relative to shVC cells (light grey fill). Pie charts show data as the mean ± s.e.m. of n = 3. * indicates significance with a p value of less than 0.05. (B) Cytospins of shVC and shCSN5 cells were stained by Jenner-Giemsa day 4 post transfection. (C) Cytospins were immunostained for tubulin (FITC) and Hoescht (blue) for visualisation of DNA in shVC and shCSN5 cells day 4 post transfection. All images shown are representative of n = 3 transfections.

Cell cycle analyses revealed striking differences between the profiles of CSN2 and CSN5 knockdown cells. Despite leading to significantly reduced cell proliferation, loss of CSN2 did not affect the cell cycle distribution of K562 cells as compared to shVC K562 cells by day 6 post transfection (Fig. 4A). Given the significantly reduced cell numbers in CSN2 knockdown by day 6, this would suggest that these cells are growing more slowly, rather than arresting at any stage in the cell cycle. In contrast, CSN5 knockdown resulted in a reduction in cells in G1 (25.7% compared to shVC 35.5%, P = 0.0044), loss of cells from S phase (21% compared to shVC 45.7%, P = 3.4 × 10^-5^) and accumulation in G2/M (33.5% compared to shVC 15.7%, P = 0.0011) by day 4 post transfection (Fig. 4A). Morphological analysis of Jenner-Giemsa stained CSN5 knockdown cells identified a significant number of large cells which appeared to be arrested in late mitosis with chaotically organized condensed chromosomes (Fig. 4B). Cells of these morphologies were not observed in either shVC (Fig. 4B) or CSN2 knockdown (Fig. Six A) cells. To investigate this morphology further, shCSN5 cells were immunostained for tubulin. As can be seen in Fig. 4C, shVC cells retained the ability to form a mitotic spindle and correctly aligned chromosomes at various stages of mitosis were observed. In striking contrast, cells lacking CSN5 demonstrated either aberrant or absent microtubule structures. In these cells, the condensed chromatids appeared to be either misaligned or indeed not associated with the spindle structures at all (Fig. 4C). This aberrant cellular phenotype is in accordance with cell cycle arrest at G2/M.

### Knockdown of CSN5 but not CSN2 results in apoptotic cell death

**Figure 5:**
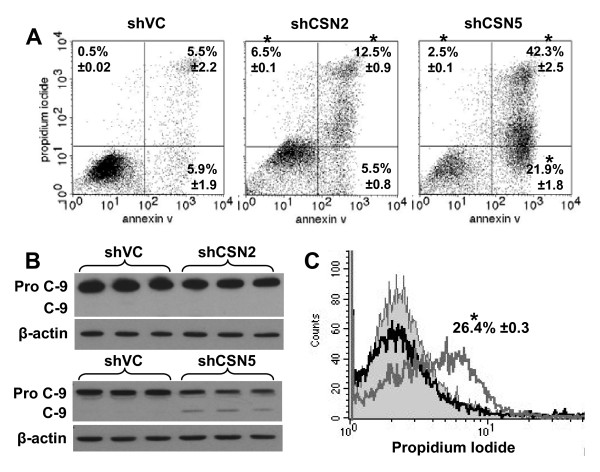
**CSN5, but not CSN2, knockdown results in apoptotic cell death**. K562 cells were transiently co-transfected with HKK plasmid together with either shVC, shCSN2 or shCSN5 plasmid. HKK positive cells were sorted 24 hours post-transfection and re-cultured. (A) Binding of Annexin V and uptake of propidium iodide were analysed by flow cytometry. The lower left quadrant encompasses the viable population of cells, the lower right quadrant contains early apoptotic cells, the upper right quadrant contains late apoptotic cells and the upper left quadrant contains the necrotic cell population. Dot plots shown are representative of n = 3 transfections. The mean of three data sets was taken and the values shown in the corresponding quadrant ± s.e.m. * indicates significance with a p value of less than 0.05. (B) Cells were harvested day 6 (shCSN2, top panels) or day 4 (shCSN5, bottom panels) post transfection and the level of caspase-9 cleavage determined by western blot. (C) Propidium iodide uptake was determined day 6 post transfection. The histogram shown is representative of n = 3 shVC (light grey in fill), shCSN2 (dark grey line) and shCSN5 (black line). The significant shift in propidium iodide staining in shCSN2 cells is shown ± s.e.m. * indicates significance with a p value of less than 0.05.

In order to determine whether the subsequent loss of viability in CSN2 and CSN5 knockdown cells was due to apoptosis, cells were co-analysed for annexin V staining and propidium iodide uptake day 7 and day 6 post transfection, respectively (Fig. 5A). Annexin V binds phosphatidylserine, a phospholipid which is translocated from the inner to the outer leaflet of the plasma membrane during early apoptosis. Propidium iodide is taken up by all cells but is efficiently effluxed by viable cells, whereas dead/dying cells remain PI-positive. In accordance with the marked increase in the sub-G1 fraction of shCSN5 cells (Fig. 4A), knockdown of CSN5 lead to significant increases in both early apoptotic (annexin V^+ve^:PI^-ve^) (21.9 ± 1.8%, P = 0.0015) and late apoptotic (annexin V^+ve^:PI^+ve^) cells (42.3 ± 2.5%, P = 0.0006) compared to shVC cells (5.9 ± 1.9% and 5.5 ± 2.2%, respectively; Fig. 5A). Furthermore, CSN5 knockdown cells demonstrated cleavage of caspase 9 (Fig. 5B), consistent with death by apoptosis in these cells. In marked contrast, CSN2 knockdown showed only a relatively small increase in annexin V positive cells (5.5 ± 0.8% and 12.5 ± 0.9%, respectively; Fig. 5A) and no caspase 9 activation (Fig. 5B). Instead, the CSN2 knockdown cells demonstrated increased retention of PI in the annexin V^-ve ^cell population (Fig. 5C, P = 3.3 × 10^-5^). This large shift in PI single-positivity relative to shVCs was not observed in CSN5 knockdown cells (Fig. 5A &5C). Thus knockdown of CSN2 in K562 cells did not result in overt apoptosis. Taken together these data indicate that knockdown in K562 cells of CSN5, but not CSN2, resulted in apoptotic cell death and that loss of cell viability in the CSN2 knockdowns was likely to be by a non-apoptotic mechanism.

### CSN2 but not CSN5 knockdown is associated with autophagy

**Figure 6:**
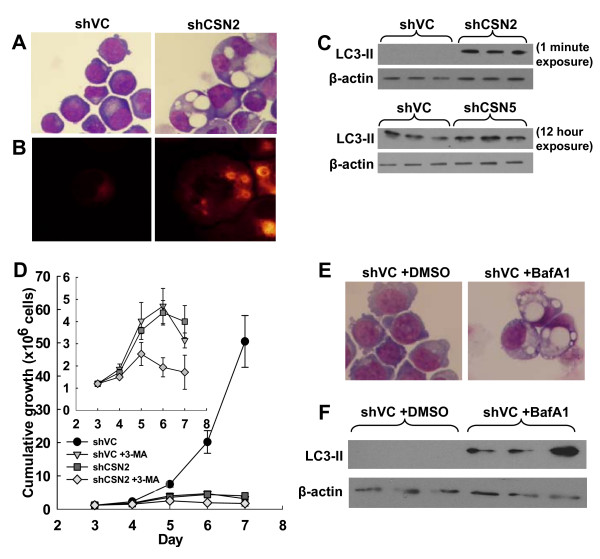
**Cells lacking CSN2, but not CSN5, are associated with autophagy**. K562 cells were transiently co-transfected with HKK plasmid together with either shVC, shCSN2 or shCSN5 plasmid. HKK positive cells were sorted 24 hours post-transfection and re-cultured. (A) Jenner-Giemsa staining of shVC and shCSN2 cytospins day 6 post transfection. (B) shVC and shCSN2 cells were stained with the autophagosome marker monodansylcadaverine day 6 post transfection. All images shown are representative of n = 3 transfections. (C) Cells were harvested day 6 (shCSN2, top panels) or day 4 (shCSN5, bottom panels) post transfection and the level of LC3-II protein determined by western blot. Even loading was determined by β-actin signal. (D) Cell counts were taken daily and the cumulative growth of shVC cells and shCSN2 cells +/- 3-MA calculated. The insert has had the untreated shVC data removed in order to demonstrate the similarity between the shVC +3-MA and shCSN2 cumulative growth profiles. Data shown is the mean of n = 3 ± s.e.m. (E) Jenner-Giemsa staining of shVC +/- 1 μM bafilomycin A1 cytospins day 7 post transfection. Images shown are representative of n = 3 transfections. (F) Cells were harvested day 7 post transfection and the level of LC3-II and β-actin protein determined by western blot.

Whilst culturing the transfectants, marked differences in cell morphology were noted in the CSN2 knockdown cells. Jenner-Giemsa staining of cytospins identified large vacuoles in a significant proportion of CSN2 knockdown cells which were not present in either shVC or shCSN5 cells (Fig. 6A). Staining with the autophagosome/autophagic vacuole marker monodansylcadaverine (MDC, [35]) identified these large vacuoles as autophagic bodies (Fig. 6B). Moreover, western blotting identified a highly significant accumulation of the autophagy associated LC3-II protein [36] in CSN2 knockdown cells relative to shVC cells (Fig. 6C). In contrast, no increase of LC3-II was detected in CSN5 knockdown cells. To demonstrate this we used a prolonged exposure (12 hours) of autoradiographs that allowed detection of basal expression of LC3-II in shVC cells and demonstrated that this was not increased in shCSN5 knockdown cells (Fig. 6C). Thus, knockdown of CSN2, but not CSN5, appeared strongly associated with autophagy. However, LC3-II accumulation can occur as a result of either enhanced autophagy or inhibition of autophagy dependant LC3-II degradation [37]. Indeed, treatment of vector control cells with the autophagy inhibitor 3-methyladenine (3-MA) resulted in a growth pattern almost identical to that of shCSN2 cells, whilst having a relatively small additional effect on shCSN2 cells (Fig. 6D). Furthermore, treatment of vector control cells with the late stage autophagy inhibitor bafilomycin A1 recapitulated both the vacuolar morphology (Fig. 6E) and the LC3-II protein accumulation (Fig. 6F) observed in cells lacking CSN2 (Fig. 6A &6C). These observations suggest that CSN2 knockdown is associated with the inhibition of autophagy.

### Loss of CSN2 results in the formation of an alternative CSN5 containing complex

**Figure 7:**
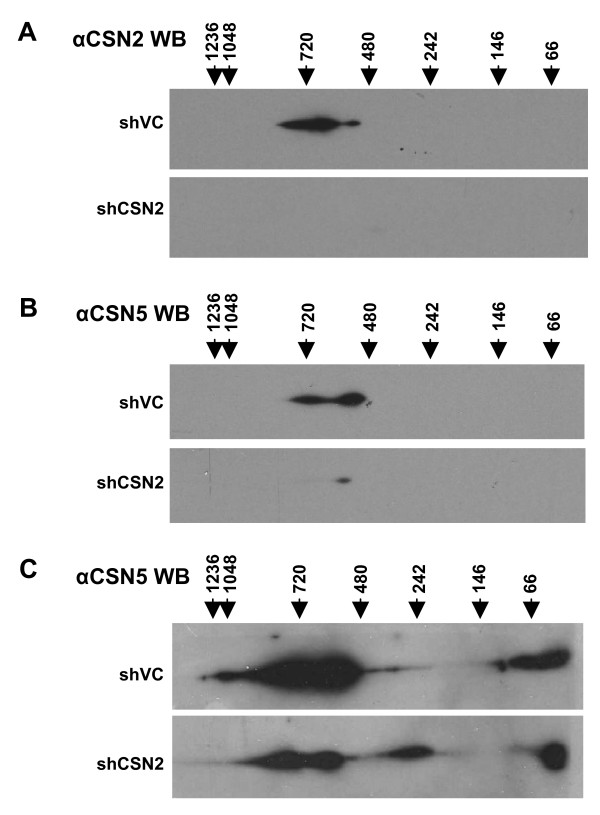
**Loss of CSN2 results in the formation of CSN5 containing CSN subcomplexes**. K562 cells were transiently co-transfected with HKK plasmid together with either shVC or shCSN2 plasmid. HKK positive cells were sorted 24 hours post-transfection, re-cultured and harvested day 3 post transfection. Both CSN2 (A) and CSN5 (B, short exposure; C, long exposure) distribution in shVC and shCSN2 cells was determined by 2-Dimensional Native-PAGE/SDS-PAGE and western blot analysis. All data shown is representative of n = 3 transfections.

The above data indicate that knockdown of CSN2 or CSN5 in K562 cells results in common derangement of SCF activity as measured by accumulation of hyperneddylated Cul1, accumulation of p27 and the sequential loss of Skp-2, cdc4 and β-TrCP. However, the cellular responses to CSN2 or CSN5 knockdown were markedly different. Since it has previously been shown that depletion of individual subunits can destabilize the CSN holocomplex [38-40] we reasoned that one explanation for the differences between CSN2 and CSN5 knockdown cellular responses may be due to the generation, in CSN2 knockdowns, of CSN5 containing CSN subcomplexes with altered activities. To investigate this, 2-dimensional native gel electrophoresis was performed on knockdown cells day 3 post transfection (Fig. 7).

Immunoblotting of extracts from shVC cells with either CSN2 or CSN5 antibody identified 2 major complexes, one of approximately 500 KDa, correlating in size with the CSN holocomplex, and the second of 750 KDa (Fig. 7A &7B). A multitude of proteins have been shown to associate with CSN subunits [41] and larger complexes have previously been observed. Monomeric CSN2 and CSN5 were also observed in the shVC cell extracts (Fig. 7C and data not shown). Following knockdown of CSN2, CSN2 protein was no longer detectable (Fig. 7A). CSN5 protein, as also shown in Fig. 1C, was greatly reduced in cells lacking CSN2, with significant loss of the CSN complex relative to vector controls (Fig. 7B). Interestingly, a longer exposure of the autoradiograph identified a CSN5 containing subcomplex of ~242 KDa in cells lacking CSN2 (Fig. 7C).

## Discussion

The achievement of almost complete CSN2 and CSN5 knockdown in this study has provided a powerful tool to study the function of these CSN subunits more closely. At the molecular level, CSN2 and CSN5 knockdowns resulted in aberrant SCF activity, with the accumulation of neddylated Cul-1, loss of the F-box protein Skp2 and an increase in the Skp2 target protein, p27. This complements another report in which CSN4 and CSN5 knockdown also resulted in increased neddylation of Cul-1 with a concomitant loss of Skp2 and increase in p27 protein in human epithelial cell lines rather than haemopoietic cells [33]. Thus it appears that the CSN complex has highly conserved activities across cells from different cell lineages and that disruption of the complex by loss of any subunit causes derangement of these activities.

It was also observed that CSN2 knockdown not only results in loss of CSN5 protein but also results in a significant reduction of CSN5 mRNA. Moreover, both CSN2 and CSN5 knockdown resulted in temporal alterations of F-box protein mRNA. Together with other recent reports [32,42], this data suggests that CSN subunits or the CSN complex as a whole may have a direct role in transcriptional regulation of CSN subunits and F-box proteins. However, it is also possible that the altered mRNA levels observed are due to secondary effects of aberrant CRL mediated protein degradation such as accumulation of proteins involved in transcriptional regulation.

Here we show for the first time sequential loss of F-box proteins following knockdown of CSN subunits. Moreover, protein levels were at least partially restored in both knockdowns upon treatment with the proteasome inhibitor MG132. These observations are in accordance with the finding that F-box proteins are autocatalytically degraded in the presence of hyperneddylated Cul-1 [28]. However, the three f-box proteins studied were each lost at a different rate, with the loss of Skp2 protein being the most rapid. The sequential loss of F-box proteins is of great interest as it may explain published results which document the loss of particular F-box proteins at a specific time point post CSN manipulation, but no reduction in other F-box proteins [28,32].

The CSN5 knockdown cultures contained aberrantly large cells and were associated with G2/M arrest and apoptosis. This data complements previous studies demonstrating that CSN5 loss inhibits proliferation and induces apoptosis [43-45]. Closer analysis of CSN5 knockdown cells identified disorganized condensed chromatids and abnormal mitotic spindles in the large cells. A recent report described stabilization of the microtubule end-binding protein 1 (EB1) by the CSN complex in human cells [46]. EB1, which is a master regulator of microtubule dynamics, was shown to bind the CSN via CSN5, and was also shown to be reduced in cells lacking CSN1 or CSN3 [46]. EB1 has recently been shown to directly interact with and regulate the activity of Aurora B, one essential component of the chromosomal passenger complex that is required for correct chromosomal alignment and spindle assembly checkpoint [47,48]. Furthermore, the dynamic behaviour of Aurora B on mitotic chromosomes has been shown to be regulated by a Cul3 E3 ligase [48]. Given that we observed hyperneddylated Cul3 in the CSN5 knockdown cells (data not shown), our data suggests that CSN5/the CSN complex is integral to the regulation of multiple components of the mitotic machinery.

K562 cells in which CSN2 had been knocked down did not display apoptosis markers as in CSN5 knockdown cells, but were instead associated with features of autophagy. Interestingly, autophagy inhibitors recapitulated the cell growth kinetics, vacuolar morphology and LC3-II accumulation of cells lacking CSN2, whilst treatment of CSN2 knockdown cells with one of these inhibitors (3-MA) had a comparatively mild effect on cell growth. These findings suggest that CSN2 knockdown K562 cells undergo autophagy inhibition resulting in non-apoptotic cell death. This is the first data to show an association between the CSN complex and autophagy.

The distinct phenotypes observed between CSN2 and CSN5 knockdowns may arise as a result of aberrant CSN5 activity within the observed CSN subcomplex in cells lacking CSN2. However, it is important to note that both the CSN subunits studied here have CSN independent functions [49-54], and that CSN5 has been shown to function within a CSN subcomplex in K562 [55]. Therefore, we cannot rule out the possible contribution of the independent functions of CSN2 and CSN5 to the phenotypic differences observed between the knockdowns. It is also noteworthy that the subcomplex observed in this study may be a result of CSN complex breakdown in the absence of CSN2 [54], rather than a functional complex contributing to the observed phenotypic differences between knockdowns. Moreover, as we see no effect of CSN5 loss on the level of CSN2 protein, one possibility not investigated here is the formation of a CSN2 containing subcomplex in the absence of CSN5. This is an intriguing possibility, particularly given the recent findings of Su et al who demonstrated an increase in the proportion of CSN2 residing in mini-complexes upon CSN8 knockdown [32]. It will be of great interest to determine the precise mechanism accounting for the divergent phenotypes encountered here, and is something which is currently under investigation.

## Conclusion

In conclusion, we have shown that loss of either CSN2 or CSN5 in human K562 cells results in significant loss of viability but by very different mechanisms, potentially attributable to the formation of a CSN5 containing subcomplex in the absence of CSN2. Furthermore, we have provided data to suggest a possible function of the CSN complex in the transcriptional regulation of both its own components and CRL subunits. Finally, we have demonstrated here for the first time the sequential loss of F-box proteins in the absence of the CSN complex and have provided the first evidence of a link between the CSN complex and autophagy.

## Methods

### Cell culture and treatments

K562 cells were cultured in RPMI 1640 supplemented with 100 U/ml penicillin, 100 μg/ml streptomycin and 10% v/v foetal bovine serum (Invitrogen, Gibco) and maintained at 37°C with 5% CO_2_. For proteasome inhibition, cells were treated with 10 μM MG132 for the final 18 hours of culturing. For autophagy inhibition, cells were treated in culture with either 10 mM 3-methyladenine from day 3-day 7 post transfection or 1 μM bafilomycin A1 for 48 hours day 5-day 7 post transfection.

### shRNA constructs

The shRNA vector used was a modified pcDNA3.1 vector (pcDNA3.1-H1) developed by Heiko Lickert [56] (kind gift from Heiner Schrewe) in which the CMV promoter has been replaced by the human RNAse P RNA H1 promoter [56]. CSN2 and CSN5 silencing sequences were selected using a siRNA design tool available on  designer.aspx and cloned into the Asp718 and XbaI restriction enzyme sites of pcDNA3.1-H1. The target sequences are as follows:

CSN2 knockdown 5'AAGCGGCATTAAGCAGTTTCC3'

CSN5 knockdown – 5'AAGGGCTACAAACCTCCTGAT3'

shRNA scramble control – 5'AAGCGGGATTCAGTAGTTACG3'

### Transfections and cell sorting

Transfection efficiencies in K562 cells vary between 20–50%. Therefore, to allow enrichment of transfected cells, 5 × 10^6 ^K562 cells were electroporated in Nucleofector kit V solution (Amaxa) using a Nucleofector I (Amaxa) and file T16, with 5 μg pMACS K^k^.II and 10 μg of the relevant knockdown pcDNA3.1-H1 plasmid according to manufacturer guidelines. The pMACS K^k^.II produces a truncated murine MHC class I cell surface protein, H-2K^k^, which lacks the cytoplasmic domain and is transiently expressed on the cell surface of transfected cells between 6 and 48 hours post-transfection. Transfected cells were sorted 24 hours post transfection using anti-H-2K^k ^antibody conjugated to magnetic beads, MACS MS columns and a MACS magnet (Miltenyi Biotec) according to manufacturer instructions. Post sorting, cells were set at 3 × 10^5^/ml daily and cells harvested for protein and mRNA analysis as indicated in results.

### Thymidine incorporation assay

2 × 10^4 ^cells were pulsed with 2 μCi/ml ^3^H-thymidine (Amersham) for the final 18 hours of culture leading up to each time point. Samples were transferred to a filter mat (Wallac) using a Skatron cell harvester (Skatron Instruments) and read using a beta-plate scintillation counter (Skatron Instruments).

### Immunofluorescence and Jenner-Giemsa staining

Cytospins were made with 5 × 10^4 ^cells in 80 μl, using a Shandon cytospin 3 (Shandon). For immunofluorescence staining, cytospins were fixed in 4% paraformaldehyde and stained using anti-β-tubulin antibody (Sigma, 1/500 dilution) followed by FITC labelled secondary antibody (Jackson Laboratories, 1/500 dilution). DNA was counterstained using Hoescht 33342 (Sigma, 1/1000 dilution). All reagents were diluted in PBS and slides mounted using Mowiol (6 g glycerol, 2.4 g Moviol-4-88 (Sigma), 12 ml 0.2 M Tris HCl pH8.5, anti-fade crystal (Sigma), 6 ml distilled water). Slides were viewed using an Axioskop2 microscope (Zeiss) and images captured with a Q-imaging 12-bit QICAM (Media Cybernetics) and Openlab software (Improvision).

For Jenner-Giemsa staining, cytospins were air-dried, methanol fixed and stained; First with Jenner staining solution (VWR, UK) diluted 1/3 in 1 mM sodium phosphate buffer pH5.6 (5 mins) and second with Giemsa stain (VWR, UK) diluted 1/20 in 1 mM sodium phosphate buffer pH5.6 (10 mins). Slides were dried and then mounted onto coverslips using DePex (VWR, UK). Slides were viewed with an Olympus BX40 microscope (Olympus) and images captured using an Olympus Chameleon digital SLR (Olympus).

### Staining of autophagosomes

For visualisation of autophagic vacuoles, 5 × 10^4 ^cells were incubated with 0.05 mM monodansylcadaverine (MDC, Sigma) in 0.5 ml PBS for 10 minutes at 37°C. Cells were washed four times with PBS, cytospins made as above and cells viewed immediately using a Leica DMIRE2 system.

### Fluorescence flow cytometry

For Annexin V labelling, 1 × 10^5 ^cells were stained using Annexin V-FITC Apoptosis Detection Kit I (BD Biosciences) according to manufacturer instructions, and staining analysed within 1 hour by flow cytometry. For cell cycle analysis 1 × 10^5 ^cells were resuspended in cell cycle buffer (10 μg/ml propidium iodide, 0.1 mM sodium chloride, 1% Triton X100) and samples analysed within 24 hours by flow cytometry. All staining was analysed using a FACS Calibur (Becton Dickinson) and the data evaluated using Cell Quest Pro software (Becton Dickinson).

### Western blot analysis

Whole cell lysates were prepared using RIPA buffer (1% v/v NP40, 0.5% w/v sodium deoxycholate, 0.1% w/v 10% SDS, in distilled water) and protein quantified using the D_c _protein assay according to manufacturer instructions (Bio-Rad). Forty micrograms of protein were boiled for 10 minutes in 1× SDS gel loading buffer (15.6 mM Tris HCl pH6.8, 6.25% v/v glycerol, 0.5% SDS, 1.25% v/v 2-mercaptoethanol, Bromophenol Blue, in distilled water). Proteins were separated by SDS-PAGE and transferred to PVDF membrane (Millipore). For western blot analysis, the following antibodies were used at 1:1000 dilution: CSN2 (Bethyl), CSN5 (Bethyl), Cul-1 (Zymed), Skp2 (Zymed), p27 (Santa Cruz), caspase-9 (Cell Signalling), LC-3 (Novus Biologicals) and β-actin (Sigma). Proteins recognized by these antibodies were detected using ant-mouse (Sigma, 1/1000 dilution) or anti-rabbit (Pierce, 1/1000 dilution) HRP conjugated secondary antibody followed by enhanced chemiluminescence (SuperSignal West Pico Chemiluminescent Substrate, Pierce) and autoradiography (Kodak X-Omat LS film, Sigma). Quantitative analysis of western blots was carried out using ImageJ software  and protein levels normalized by comparison to β-actin signals on the same membrane.

### 2-Dimensional gel analysis

Native protein extracts were obtained from 2.5 × 10^5 ^cells by resuspending cells in 50 μl mild lysis buffer (25% 4× NativePAGE sample buffer (Invitrogen), 1% digitonin, 10% 10× protease inhibitor, in distilled water). Extracts were separated out in the first dimension using a NativePAGE Novex Bis-Tris Gel System (Invitrogen) according to manufacturer instructions. The gel was then cut into individual lanes, proteins denatured by incubation in 1× SDS gel loading buffer and resolved in the second dimension by electrophoresis through 12.5% SDS-polyacrylamide gels. Proteins were transferred to PVDF membrane (Millipore) and immunoblotting performed as above.

### Quantitative real-time PCR analysis (QRT-PCR)

RNA was extracted using the Qiagen RNeasy kit according to manufacturer instructions and cDNA generated using 1 μg RNA, random hexamers (Promega) and Superscript II reverse transcriptase (Invitrogen). Quantitative real-time PCR was carried out using either TAQMAN or SYBR-Green based assays. For TAQMAN assays, QRT-PCR was carried out in duplicate 20 μl reactions containing 1× qPCR Mastermix Plus (Eurogentec), 20–40 ng cDNA, 18 pmoles each primer and 2.5 pmoles FAM/TAMRA dual labeled probes. For SYBR-Green assays, QRT-PCR was carried out in duplicate 25 μl reactions containing 1× Sensimix (Quantace), 20–40 ng cDNA, 9 pmoles each primer, 1× SYBR-Green solution (Quantace), 4 mM MgCl_2 _and 0.5 units UNG (Quantace). QRT-PCR was carried out on an ABI Prism 7000 sequence detector (Applied Biosystems). The following primers (Sigma Genosys) and FAM/TAMRA labeled probes (Eurogentec) were used:

CSN2, 5'-CCTCATCCACTGATTATGGGAGT-3' (forward),

5'-CATCATAATTCTTGAAGGCTTCAAAA-3' (reverse),

5'-CCCTCAAGTGCATTTTACCACCACATTCTCT-3' (probe);

CSN5, 5'-ATATCCGCAGGGAAAG-3' (forward),

5'-GGTCCTTCATCAGGAGGTTTGT-3' (reverse),

5'- TGGCGCCTTTAGGACATACCCAAAGG-3' (probe);

Skp2, 5'-CGCTGCCCACGATCATTT-3' (forward),

5'-CCATGTGCTGTACACGAAAAGG-3' (reverse);

Cdc4, 5'-ACGACGCCGAATTACATCTGT-3' (forward),

5'-ACTCCAGCTCTGAAACATTTTTAGC-3' (reverse);

β-Trcp, 5'-GAGGCATTGCCTGTTTGCA-3' (forward)

5'-TGTCCCATAATCTGATAGTGTTGTCA-3' (reverse)

18S, 5'-GCCGCTAGAGGTGAAATTCTTG-3' (forward),

5'-CATTCTTGGCAAATGCTTTCG-3' (reverse).

Preoptimised primers and probes to 18S ribosomal RNA were used as internal standards in TAQMAN QRT-PCR (Applied Biosystems). Cycle threshold (Ct) values were obtained graphically for test genes and 18S internal standards. ΔCt values were calculated by subtracting 18S Ct from test gene Ct, and average ΔCt values obtained from duplicates. Relative mRNA levels were determined by subtraction of mock transfection ΔCt values from shVC/shCSN2/shCSN5 ΔCt values to give a ΔΔCt value and conversion through 2^-ΔΔCt^.

## Authors' contributions

CP conceived of and designed the study, carried out all laboratory experimentation except for that carried out by REH and FLK, and drafted the manuscript. REH harvested thymidine plates, generated the propidium iodide flow cytometry data and assisted in the analysis of all flow cytometry data. CMB and FLK conceived of and designed the study and drafted the manuscript. FLK designed the shRNA sequence and generated the plasmid for CSN2 knockdown. All authors read and approved the manuscript.

## Supplementary Material

Additional file 1 **Vector control scramble sequence has no effect on protein levels, mRNA expression or cell growth, whilst CSN5 knockdown resulted in the sequential loss of F-box proteins**. K562 cells were transiently co-transfected with HKK plasmid together with either empty vector or vector control scramble plasmid. HKK positive cells were sorted 24 hours post-transfection, re-cultured and harvested day 9 post transfection. (A) The levels of CSN2, CSN5, Cul1, Skp2, p27, LC3-II and caspase-9 protein was determined by western blot. (B) CSN2 and CSN5 mRNA levels in vector control scramble cells relative to empty vector transfected cells were determined by QRT-PCR. Data is the mean of n = 3 transfections ± s.e.m. The dashed line indicates mRNA expression in empty vector transfected cells. (C) Cell counts were taken daily and the cumulative growth calculated. The cumulative growth of shVC scramble cells is shown relative to empty vector transfected cells. Data shown are the mean ± s.e.m. of n = 3. (D) shVC and shCSN5 cells were harvested day 4 post transfection and the level of Skp-2, Cdc4, β-TrCP and β-actin protein determined by western blot. Click here for file
