# Supplementary figures and images for: Analysis of the role of COP9 Signalosome (CSN) subunits in K562; the first link between CSN and autophagy
Source: BMC Cell Biol. 2009 Apr 28;10:31. doi: 10.1186/1471-2121-10-31 (PMC2685372; doi:10.1186/1471-2121-10-31)

## Supplementary Figure 1

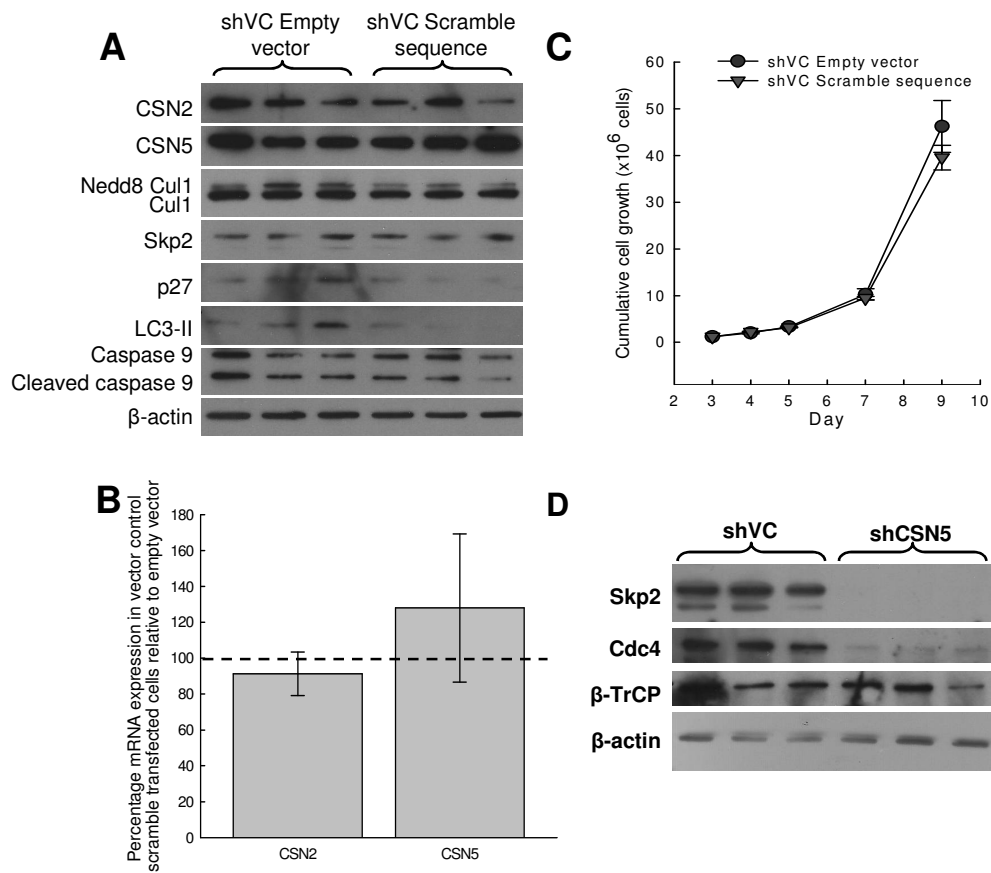

Supplement: Additional file 1 — Vector control scramble sequence has no effect on protein levels, mRNA expression or cell growth, whilst CSN5 knockdown resulted in the sequential loss of F-box proteins. K562 cells were transiently co-transfected with HKK plasmid together with either empty vector or vector control scramble plasmid. HKK positive cells were sorted 24 hours post-transfection, re-cultured and harvested day 9 post transfection. (A) The levels of CSN2, CSN5, Cul1, Skp2, p27, LC3-II and caspase-9 protein was determined by western blot. (B) CSN2 and CSN5 mRNA levels in vector control scramble cells relative to empty vector transfected cells were determined by QRT-PCR. Data is the mean of n = 3 transfections ± s.e.m. The dashed line indicates mRNA expression in empty vector transfected cells. (C) Cell counts were taken daily and the cumulative growth calculated. The cumulative growth of shVC scramble cells is shown relative to empty vector transfected cells. Data shown are the mean ± s.e.m. of n = 3. (D) shVC and shCSN5 cells were harvested day 4 post transfection and the level of Skp-2, Cdc4, β-TrCP and β-actin protein determined by western blot. [file 1471-2121-10-31-S1.pdf]
